# Skin Sensitization Evaluation of Carbon-Based Graphene Nanoplatelets

**DOI:** 10.3390/toxics9030062

**Published:** 2021-03-17

**Authors:** Sung-Hyun Kim, So-Hye Hong, Jin Hee Lee, Dong Han Lee, Kikyung Jung, Jun-Young Yang, Hyo-Sook Shin, JeongPyo Lee, Jayoung Jeong, Jae-Ho Oh

**Affiliations:** Division of Toxicological Research, National Institute of Food and Drug Safety Evaluation, Ministry of Food and Drug Safety, Osong, Cheongju 28159, Korea; tjdgus32@korea.kr (S.-H.K.); shhong99@korea.kr (S.-H.H.); tod98@korea.kr (J.H.L.); donghan04@korea.kr (D.H.L.); kikyung@korea.kr (K.J.); yangjy@korea.kr (J.-Y.Y.); aqua978@korea.kr (H.-S.S.); origene@korea.kr (J.L.); 0jjy@korea.kr (J.J.)

**Keywords:** skin sensitization, alternative to animal testing, KeratinoSens^TM^, local lymph node assay (LLNA), nanomaterial, graphene

## Abstract

Graphene nanoplatelets (GNPs) are one of the major types of carbon based nanomaterials that have different industrial and biomedical applications. There is a risk of exposure to GNP material in individuals involved in their large-scale production and in individuals who use products containing GNPs. Determining the exact toxicity of GNP nanomaterials is a very important agenda. This research aimed to evaluate the skin sensitization potentials induced by GNPs using two types of alternative to animal testing. We analyzed the physicochemical characteristics of the test material by selecting a graphene nanomaterial with a nano-size on one side. Thereafter, we evaluated the skin sensitization effect using an in vitro and an in vivo alternative test method, respectively. As a result, we found that GNPs do not induce skin sensitization. In addition, it was observed that the administration of GNPs did not induce cytotoxicity and skin toxicity. This is the first report of skin sensitization as a result of GNPs obtained using alternative test methods. These results suggest that GNP materials do not cause skin sensitization, and these assays may be useful in evaluating the skin sensitization of some nanomaterials.

## 1. Introduction

Graphene nanoplatelets (GNPs), a two-dimensional monocrystalline layer form of carbon, are a major type of carbon-based nanomaterial which are used for various industrial and biomedical applications. Graphene has drawn attention across a vast field, such as in diverse devices or applied in batteries [1,2]. In recent years, as the types and production of GNPs have increased, concerns about toxicity caused by human exposure have increased exponentially. The major routes of exposure for nanomaterials are ingestion, inhalation and skin penetration in the workplace. Skin penetration of nanomaterials can induce lesions, contact allergy, local inflammation, and skin sensitization [3,4]. However, there are no studies on the toxicological database for GNP in skin sensitization which is the most easy exposure route in the workplace.

In addition, with the recent exponential increase in commercialization of nanomaterials for use in the cosmetic industry, etc., and the safety concerns associated with these, Nano safety evaluation has gained importance [5]. The focus on alternative methods in cosmetic testing is also increasing due to concerns regarding animal welfare and the 3Rs principle of replacement, reduction, and refinement [6,7]. However, as these guidelines are based on chemical substances, there is a need to develop alternative test methods that reflect the properties of nanomaterials.

The Organization for Economic Cooperation and Development (OECD) has suggested an adverse outcome pathway (AOP) leading to allergic contact dermatitis (ACD) starting with a molecular initiating event [8]. This shows that the related chemical and biological mechanisms can induce skin sensitization through a total of four key events. In general, the AOP of skin sensitization are classified by non-animal or animal testing methods. Animal-free test methods include the following assays: Direct Peptide Reactivity Assay (DPRA, key event 1), an in chemico assay, the antioxidant/electrophilic element (ARE) based Nuclear factor-erythroid 2-related factor 2 (Nrf2) luciferase test using a keratinocyte cell line (KeratinoSens^TM^ assay, key event 2), and human cell line activation test (h-CLAT, key event 3) that simulates dendritic cells [9,10,11]. On the other hand, the key event 4 test method to confirm the activation of T cells is an alternative using experimental animals. Known as local lymph node assay (LLNA), this stage can be used to confirm activation of lymph nodes by sensitizers using enzyme-linked immunosorbent assay (ELISA) or flow cytometry (FCM) [12].

Evaluation of several nanomaterials’ skin sensitization was recently performed and the applicability of these assays for testing nanomaterials was evaluated [13,14]. However, a lack of information regarding skin sensitization due to various nanomaterials, including GNPs, remains. Therefore, we evaluated the skin sensitization potential of GNPs using both alternative test methods, ARE-Nrf2 Luciferase KeratinoSens^TM^ and LLNA: BrdU-FCM assays.

## 2. Materials and Methods

### 2.1. Graphene Nanoplatelets

GNP (CAT# 06-0225) materials were purchased from Strem Chemicals (Newburyport, MA, USA). Morphology of GNPs was observed by transmission electron microscopy (TEM; JEM-1200EX II, JEOL, Tokyo, Japan) and zeta potential was measured using a Zetasizer-Nano ZS instrument (Malvern Instruments, Malvern, UK) in different working solutions: Dulbecco’s Modified Eagle’s Medium (DMEM; GIBCO, Grand Island, NY, USA) containing 1% heat-inactivated fetal bovine serum (FBS; GIBCO) and N,N-Dimethylformamide (DMF; CASRN. 68-12-2, Sigma-Aldrich, St Louis, MO, USA) solution containing 3% heat-inactivated mice serum. Endotoxin were evaluated using an Endpoint Chromogenic Limulus Amoebocyte Lysate QCL-1000 (CAT# 50-647U) assay (Cambrex, Walkersville, MD, USA). The endotoxin of GNP was measured according to the method and procedure provided by the kit manufacturer.

### 2.2. Preparation of GNP Suspensions

GNP suspensions were prepared by slightly modifying a formerly described method [15]. Briefly, GNP stock solutions were dispersed in distilled water (DW) and sonicated at 40 KHz with 100 W output power for 30 min in a bath sonicator (Saehan-Sonic, Seoul, Korea). Thereafter, fresh DMEM media supplemented with 1% FBS was added to different working concentrations (0.98–2000 µM). Since re-aggregation of GNP may be induced when DMEM culture medium is added, ultrasonic dispersion was performed for an additional 30 min. In LLNA: 5-bromo-2′-deoxyuridine (BrdU)-FCM assay, GNP stock solution was dispersed in DW and sonicated by the same procedure. Thereafter, serum 3% of the final volume was added to the dispersed stock solution and further dispersed for 30 min. Finally, since re-aggregation of GNP may be induced when DMF solution (working concentration: 25, 50, and 100% *v*/*v*) is added, ultrasonic dispersion was performed for an additional 30 min.

### 2.3. KeratinoSens^TM^ Cell Culture

A transgenic human keratinocyte cell line, with a stable insertion of the Luciferase reporter gene under control of the ARE-element KeratinoSens^TM^ were provided by Givaudan Suisse SA (Vernier, Switzerland). KeratinoSens^TM^ were cultured in DMEM media supplemented with 10% FBS, 0.5 mg/mL Geneticin (Sigma-Aldrich). The cells were sub-cultured every 2–4 days at 80–90% confluence for a maximum of 25 passages. Stabilized KeratinoSens^TM^ were seeded into 96-well cell culture plate at a density of 10,000 cells/well. Cell cultured plates were incubated in a humidified atmosphere condition of 5% CO_2_ at 37 °C.

### 2.4. GNP Suspension Treatments and KeratinoSens^TM^ Assay Methods

KeratinoSens^TM^ were incubated overnight to reach approximately 80% confluency. The cells were washed once with pre-warmed pH 7.4 DPBS (Gibco), followed by the addition of dispersed GNPs suspension (0.98–2000 µM), and the culture plates were then incubated for 48 h. Positive control, cinnamic aldehyde (CASRN. 14371-10-9, Sigma-Aldrich), was tested in parallel (concentration: 4–64 µM). The viability of the treated KeratinoSens^TM^ was measured using the thiazolyl blue tetrazolium bromide (3-(4,5-dimethylthiazo-2-yl)-2,5-diphenyl-tetrazolium bromide assay reduction test (Promega, Madison, WI, USA). To exclude colorimetric interference from nanomaterials present in the cells, the supernatant was transferred into clear 96-well plates and the absorbance was measured at 570 nm with a microplate reader (Tecan, Männedorf, Switzerland). The cell viability (%) was calculated based on the optical density of the vehicle control and blank control. Then, to measure the luciferase activity of GNP, we used the One-Glo^TM^ Luciferase assay kit (Promega). The luciferase assay was conducted under the same conditions as the MTT assay. The luminescence intensity of each sample was measured using a multi-microplate reader (Synergy 2, BioTek, Winooski, VT, USA). Level of luciferase induction was calculated based on the luminescence values of the vehicle control and blank control.

### 2.5. Pro-Inflammatory Cytokines Analysis of Supernatant

In order to analyze inflammatory factors after exposure of GNPs to keratinocytes, the supernatant was separated and measured for inflammatory cytokines. The samples used were treated with a GNP solution for 48 h using the same procedure as for the KeratinoSens^TM^ test, and then measured at concentrations of 500, 1000, and 2000 μM. The levels of inflammatory cytokines including interleukin (IL) -1α, IL-1β, IL-8, and tumor necrosis factor- (TNF) -α were measured using commercially available ELISA kits. All ELISA Kits were purchased from R&D systems (Duoset kit, Minneapolis, MN, USA) and measured according to the manufacture’s procedure.

### 2.6. Animals

Female, seven weeks old, BALB/C mice (Specific Pathogen Free, SPF) were purchased from ORIENT BIO Inc (Seongnam, Korea). Mice were kept at an animal facility in the Ministry of Food and Drug Safety (MFDS), Korea and acclimated for at least six days before the experiments. Mice were housed in a relative humidity of 30–70% at 22 ± 3 °C. Food and water were supplied ad libitum. This experiment was approved by the Institutional Animal Care and Use Committee (IACUC) of MFDS (Approval number: MFDS-20-013c2; date: 23 April 2020).

### 2.7. GNP Treatments and LLNA: BrdU-FCM Assay Methods

On days 1, 2, and 3, dispersed GNPs suspension (working concentration: 25, 50, and 100% *v*/*v*, respectively), vehicle control (DMF contained 3% mouse serum), and positive control (25% hexyl cinnamic aldehyde), were applied to the dorsal skin of each ear of the mouse at the same time-point (group per four). Acetone:olive oil (4:1 *v*/*v*, AOO) solvent was used to prepare the positive control based on the OECD test guideline 442B. GNP suspension was prepared fresh daily before application. On day 5, all mice were intra-peritoneally (i.p) injected with 100 μL of BrdU solution (20 mg/mL). On day 6, mice were sacrificed and their auricular lymph nodes were isolated. Excised lymph nodes were mashed using a spatula to prepare lymph node cells (LNCs). LNCs were counted using a hemocytometer after staining with trypan-blue (Sigma). The quantitated LNCs (1.5 × 10^6^ cells/mL) were prepared for the LLNA: BrdU-FCM assay, according to the protocol provided in the BD Pharmingen^TM^ FITC BrdU Flow Kit (BD Biosciences, San Jose, CA, USA). Viable LNCs were counted using a BD FACS Calibur^TM^ flow cytometer (BD Biosciences) and a total of 10,000 gated cells were analyzed, as previously described [16,17]. Stimulation index (SI) were calculated using the formula described in the OECD test guideline 442B [12]. If the SI was 2.7 or greater, the test materials were classified as sensitizers.

$$Stimulation\;Index\;\left(SI\right)=\frac{\mathrm{Number}\;\mathrm{of}\;\mathrm{BrdU}-\mathrm{positive}\;\mathrm{LNCs}/\mathrm{mouse}\;\mathrm{exposed}\;\mathrm{to}\;a\;\mathrm{test}\;\mathrm{material}}{\mathrm{Mean}\;\mathrm{number}\;\mathrm{of}\;\mathrm{BrdU}-\mathrm{positive}\;\mathrm{LNCs}\;\mathrm{in}\;\mathrm{the}\;\mathrm{vehicle}\;\mathrm{control}\;\mathrm{group}}$$

### 2.8. Statistical Analysis

Data were analyzed with GraphPad Prism software ver. 5.0 (La Jolla, CA, USA) and presented as mean ± standard error of the mean (SEM). Statistical analysis was performed by one-way ANOVA and each group was compared by post-hoc Turkey’s pairwise comparisons. A result of *p* value < 0.05 was considered as statistically significant.

## 3. Results

### 3.1. Physicochemical Characteristic of GNPs

**Figure 1:**
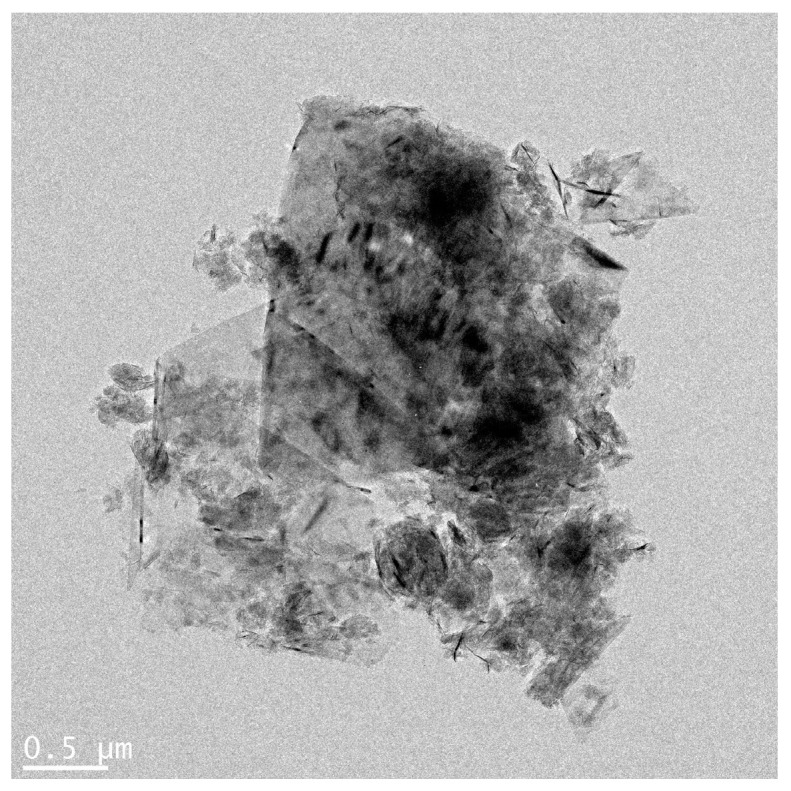
Agglomeration form of graphene nanoplatelets (bar = 0.5 µm).

Physicochemical properties of GNPs are summarized in Table 1 and Figure 1. According to the information provided by the manufacturer, the graphene nanoplatelet aggregates used are submicron platelet aggregates less than 2 microns in diameter and several nanometers thick. Measurement of the zeta potential showed that GNPs were negatively-charged, with charge in distilled water (DW) and working solution. The results of Limulus Amoebocyte Lysate test showed that GNPs had endotoxin levels that were lower than the limit of detection (0.1 U/mL).

### 3.2. Evaluation of GNPs in the KeratinoSens^TM^ Assay

**Figure 2:**
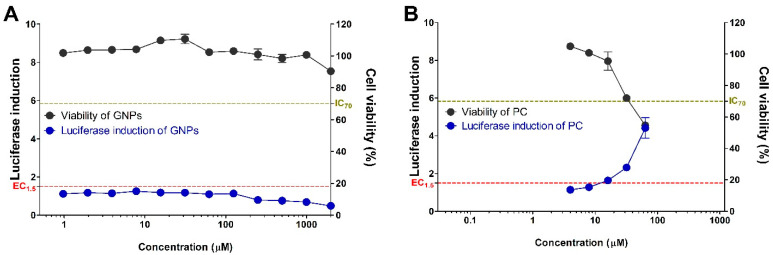
Induction of luciferase activity (blue line) and viability (black line) in the KeratinoSens^TM^ assay. The cells were treated with the (**A**) graphene nanoplatelets (GNPs), and (**B**) positive control (cinnamic aldehyde, CAS number 14371-10-9). Data are expressed as mean ± SEM (*n* = 6). Positive control was tested in parallel (4–64 µM).

In order to apply the OECD Test Guideline 442D to graphene, a proficiency test using 10 proficiency substances suggested in the guideline was performed and results presented in Table 2. After completing the verification for a total of 10 substances, the GNPs were assessed for their skin sensitization potential using the KeratinoSens^TM^ assay (Figure 2 and Table 3). GNPs did not induce the activity of the luciferase. The EC1.5 (interpolated concentration for a 1.5-fold luciferase induction) value for the GNPs was >2000 µM, thus classifying it as a non-sensitizer. IC50 (concentration effecting a reduction of cellular viability by 50%) values were found to be >2000 µM and cytotoxicity for GNPs was not found.

### 3.3. Evaluation of Pro-Inflammatory Cytokines in KeratinoSens^TM^ Cells

**Figure 3:**
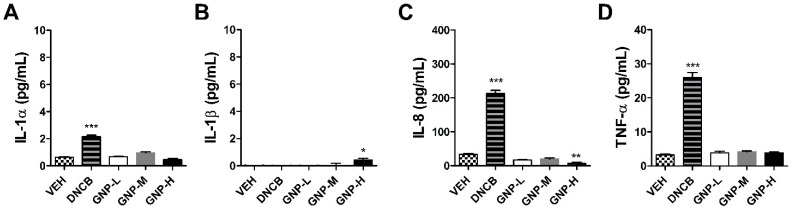
Expression of inflammatory cytokine in the KeratinoSens^TM^ cells treated with the GNPs. The parameters were as follows: (**A**) IL-1alpha, (**B**) IL-1beta, (**C**) IL-8, and (**D**) tumor necrosis factor (TNF)-alpha (VEH = vehicle control, Low-dose = 500 µM, Mid-dose = 1000 µM, High-dose = 2000 µM). Data are expressed as mean ± SEM values (*n* = 4). Each treatment group was compared with the vehicle control group to determine statistical significance. * *p* < 0.05; ** *p* < 0.005; *** *p* < 0.0001.

Figure 3 shows the inflammatory factor analysis results of KeratinoSens^TM^ cells treated with GNPs. GNPs showed a measurement result that did not exceed the range of the control group in the measured IL-1alpha, 8 and TNF-alpha cytokine results compared to the positive control group (3 µM), 2,4-Dinitrochlorobenzene (DNCB). In the IL-1beta, statistical significance was observed at high concentration (2000 μM), but this was below the detection limit of the kit, 1.95 pg/mL.

### 3.4. Evaluation of GNPs in the LLNA: BrdU-FCM Assay

**Figure 4:**
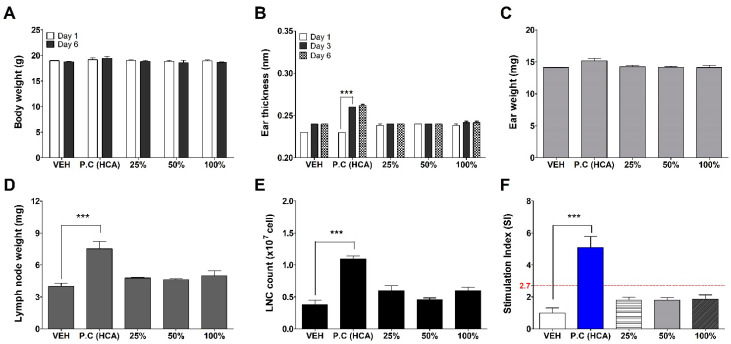
Results of GNPs skin sensitization potential in LLNA: BrdU-FCM. The evaluation parameters were as follows: (**A**) Body weight (g), (**B**) Ear thickness (mm), (**C**) Ear weight (mg), (**D**) Lymph node weight (mg), (**E**) Lymph node cell (LNC) count (×10^7^ cells), and (**F**) Stimulation Index (SI). Data are expressed as mean ± SEM (*n* = 4). Each treatment group was compared with the vehicle control group to determine statistical significance. *** *p* < 0.0001.

GNPs were assessed for potential skin sensitization using the LLNA: BrdU-FCM, in vivo assay (Figure 4). No significant results were found at any concentration of GNPs except for the positive control (25% hexyl cinnamic aldehyde) group for a total of six parameters: body weight, ear thickness and weight, lymph weight and lymph cell number, and stimulation index used for sensitization evaluation. Finally, the Stimulation Index (SI) value was found to be less than 2.7, as calculated by flow cytometry, and was judged as a non-sensitizer through criteria of test guideline 442B.

## 4. Discussion

GNPs, which have been called the ‘dream material’ have attracted attention for their extraordinary physicochemical properties, because of a wide range of promising applications in the biomedical and electronic fields [18]. Especially, GNP can be used to improve the properties of a wide range of polymeric materials, including thermoplastic and thermoset composites, natural or synthetic rubber, adhesives, thermoplastic elastomers, and paints and coatings because of their unique nanoscale size, shape, and material composition [19,20]. Since use is rapidly increasing in various industries during recent years, the safety of production workers (in workplaces) can be guaranteed through laws or regulations only when accurate identification of toxicity for the substance has been made.

**Figure 5:**
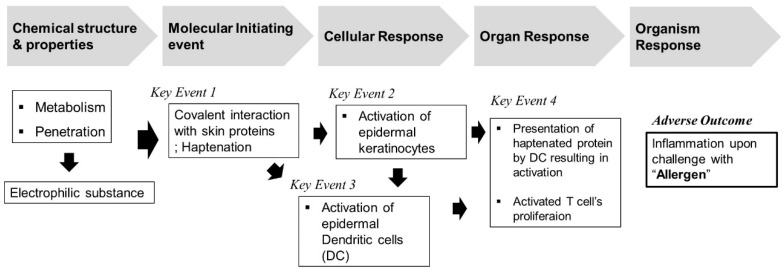
Overview of adverse outcome pathway (AOP) for skin sensitization.

Recently, with the growing emphasis on the 3Rs principle for testing, the use of animals in toxicity studies has become a major issue in the international community [6]. Alternative testing methods not involving the use of animals have been suggested by various countries and institutions, including the European Union Reference Laboratory for Alternatives to Animal Testing, the Interagency Coordinating Committee on the Validation of Alternative Methods, and the Japanese Center for the Validation of Alternative Methods. Studies are currently being carried out regarding this subject and the OECD has approved, enacted, and distributed guidelines for alternative test methods. The OECD TG 442 guidelines can be classified into four key events: key event 1: molecular initiation event, key event 2 and key event 3: cellular responses, and key event 4: organ-level responses based on AOP-inducing skin sensitization (Figure 5). These alternative test method guidelines can be evaluated on a chemical basis, but nanomaterials have the potential to act as happens due to a variety of physicochemical properties including nanoscale small sizes [21,22]. Therefore, in the current study, the test substances, GNPs, were evaluated for key event 2 and key event 4 by employing methods for confirming cellular responses and organ level responses in skin sensitization AOP.

Accuracy of the ARE-Nrf2 Luciferase KeratinoSens^TM^ assay for identifying sensitizers was determined to be 77% (155/201) with a sensitivity of 78% (71/91). Laboratory-to-laboratory reproducibility has been reported to be approximately 85% [23,24]. Although there are limitations on testing insoluble substances, some research has demonstrated that these substances can be evaluated [25,26]. The LLNA: BrdU-FCM test method employs the use of animals and previously reported studies have suggested the possibility of evaluating nanomaterials using this approach. For instance, Park et al. [13] conducted an LLNA test using titanium nanomaterials and reported that titanium does not induce skin sensitization.

In our study, GNPs are insoluble in most solvents and tend to easily form aggregates. Proper dispersion is very important for accurately predicting toxicity and hence homogeneous dispersion of the nanomaterials in solvents is important. In the current study, we used serum protein to improve the dispersion of GNPs in both the in vitro ARE-Nrf2 Luciferase KeratinoSens^TM^ test and the in vivo LLNA: BrdU-FCM test. In the in vitro test, dispersion was induced by including FBS as a component in the medium. Meanwhile, mouse serum was used as a nanomaterial dispersant in the in vivo tests [27]. This was chosen based on previous reports that inactivated serum obtained from the same species reduces the amount of large aggregation and that there are no side effects caused by serum [28,29].

In the current report, we describe for the first time sensitization results of GNPs using the ARE-Nrf2 Luciferase KeratinoSens^TM^ and LLNA: BrdU-FCM test methods. In summary, the skin sensitization results for GNPs using the two alternative tests were negative. In our study, it was shown that the well-known sensitizer-induced inflammation indicator IL-1alpha was not induced [30,31]. Moreover, when judged considering all the results such as IL-1beta, IL-8 and TNF-alpha, which are cytokines related to acute inflammatory reactions, graphene does not appear to have an inflammatory effect on keratinocytes. The IL-1beta cytokine result is very small, but the significant increase in GNPs compared to the control may have been induced by inflammasome formation induced by intracellular incomplete phagocytosis depending on the type of substance [32]. In addition, this is related to the decrease in cell viability of GNPs at the highest concentration. DNCB, a sensitizer, is a completely soluble chemical, so it does not appear to induce IL-1beta through complete phagocytosis.

Until now, there is no research on skin sensitization of graphene, but there are cases of the evaluation of carbon-based nanomaterials. Although graphene and carbon nanotubes are completely different shape materials, they are nanomaterials that have the same element as carbon. Carbon nanotubes have been reported as non-sensitizing materials through the guinea pig sensitization test and the mouse lymph node test [33,34]. In our previous study, carbon nanotubes, evaluated skin sensitization using alternative studies in vitro and in vivo and did not induced sensitization [15].

## 5. Conclusions

We report the sensitization test results of GNPs using the in vitro and in vivo alternative test methods. We found that GNPs did not induce skin sensitization in both assays. Nano-graphene used as a product exists as various types because it attaches to functional groups or is manufactured in different sizes and layers. However, the current study reports only one type of graphene evaluation result. In summary, to secure the safety of commercialized GNP, it is suggested that additional data acquisition is necessary through more research. Furthermore, it is necessary to protect workers in the workplace and establish guidelines for skin sensitization specific to nanomaterials for use in various studies.

## Figures and Tables

**Table 1:** Characteristics of graphene nanoplatelets.

GNPs	In KeratinoSens^TM^	In Local Lymph Node Assay (LLNA): BrdU-FCM
Diameter	<2 microns, and a thickness of a few nanometers	
Surface area (m^2^/g)	300	
Zeta potential (mV)		
in DW	−23.32 ± 0.80	−23.32 ± 0.80
in working solution *	−27.17 ± 0.94	−24.60 ± 0.26
Molecular weight (g/mol)	12.01	
Carbon content (%)	>98	
Endotoxin (EU/mL)	<0.1 (all samples)	

* Working solution (KeratinoSens^TM^) was prepared with DW stock (1%) + DMEM, containing 1% FBS. Working solution (LLNA: BrdU-FCM) was prepared using DW stock (10%) + DMF, containing 3% mouse serum. Data are expressed as mean ± SEM, *n* = 6; GNPs = Graphene nanoplatelets, DW = distilled water, EU = endotoxin unit, DMEM = Dulbecco’s Modified Eagle’s Medium, FBS = Fetal bovine serum, DMF = N,N-Dimethylformamide.

**Table 2:** Demonstrating technical proficiency of the KeratinoSens^TM^ test method.

Proficiency Substances	CAS RN	Physical Form	In Vivo Prediction	Reference Range *		KeratinoSens^TM^ Assay		
				EC_1.5_ (µM)	IC_50_ (µM)	EC_1.5_ (µM)	IC_50_ (µM)	Results
Isopropanol	67-63-0	Liquid	Non-sensitizer	>1000	>1000	>1000	>1000	Negative
Salicylic acid	69-72-7	solid	Non-sensitizer	>1000	>1000	>1000	>1000	Negative
Lactic acid	50-21-5	Liquid	Non-sensitizer	>1000	>1000	>1000	>1000	Negative
Glycerol	56-81-5	Liquid	Non-sensitizer	>1000	>1000	>1000	>1000	Negative
Cinnamyl alcohol	104-54-1	solid	Week-sensitizer	25–175	>1000	36.5	>1000	Positive
Ethylene glycol dimethacrylate	97-90-5	Liquid	Week-sensitizer	5–125	>500	77.2	795.6	Positive
2-Mercapto benzothiazole	149-30-4	solid	Moderate-sensitizer	25–250	>500	129.4	573.4	Positive
Methyldibromo glutaronitrile	35691-65-7	solid	Strong-sensitizer	<20	20–100	1.9	42.7	Positive
4-Methylamino phenol sulfate	55-55-0	solid	Strong-sensitizer	<12.5	20–200	7.9	48.3	Positive
2,4-Dinitro-chlorobenzene	97-00-7	solid	Extreme-sensitizer	<12.5	5–20	2.6	12.4	Positive

* Information on the reference range was provided in OECD test guideline 442D.

**Table 3:** GNPs evaluated in KeratinoSens^TM^ assay.

NMs	CAS RN	Physical Form	KeratinoSens^TM^ Assay Results				
			Imax	EC_1.5_ (µM)	Viability (%) *	IC_50_ (µM)	Classification
**GNPs**	7782-42-5	Solid	1.27	>2000	>85	>2000	Negative

* Cell viability (%) at EC1.5; NMs: Nanomaterials.

## Data Availability

The original contributions presented in the study are included in the article, further inquiries can be directed to the corresponding authors.
